# Early Life Antibiotic Exposure and Intestinal Colonization by Enterobacteriaceae upon Admission to a Neonatal Referral Unit: A Case–Control Study

**DOI:** 10.3390/antibiotics15020123

**Published:** 2026-01-27

**Authors:** Sergio Agudelo-Pérez, Gloria Troncoso, Martha Alvarez-Olmos, Maria Pineda, Adriana Moscote, María Paula Molina Pérez

**Affiliations:** 1 School of Medicine, Pediatrics Department, Universidad de La Sabana, Chía 250008, Colombia; mariapinto@unisabana.edu.co (M.P.); adrianamoto@unisabana.edu.co (A.M.); mariamolpe@unisabana.edu.co (M.P.M.P.); 2 Neonatal Unit, Fundación Cardioinfantil—LaCardio, Bogotá 110131, Colombia; gtroncoso@lacardio.org; 3 Fundación Cardioinfantil—LaCardio, Universidad del Rosario, Universidad El Bosque, Bogotá, Colombia; mialvarez1@lacardio.org

**Keywords:** antimicrobial stewardship, cross infection, newborn, infant, neonatal intensive care units, intestinal microbiota, Enterobacteriaceae infections

## Abstract

**Background/Objectives:** Intestinal colonization by Enterobacteriaceae, including extended-spectrum β-lactamase-producing (ESBL-E) and carbapenemase-producing (E-CPE) strains, is an early marker of multidrug-resistant infections in neonates, particularly those transferred from lower-complexity hospitals. This study aimed to identify factors associated with intestinal Enterobacteriaceae colonization upon admission to a level IV neonatal referral unit in Colombia, with a focus on prior antibiotic exposure. **Methods:** We conducted a retrospective case–control study, including all neonates transferred from peripheral hospitals and screened with rectal swabs at admission. Cases were neonates colonized with Enterobacteriaceae, and controls were non-colonized neonates admitted during the same period. Multivariable logistic regression models were used to evaluate three exposure dimensions: prior antibiotic use (yes/no), number of agents, and the WHO AWaRe classification. A secondary analysis was performed to assess the factors associated with ESBL-E and E-CPE colonization. **Results:** Among the 435 referred neonates, 87 (20.0%) were colonized, predominantly *Klebsiella pneumoniae* (53.6%) and *Escherichia coli* (19.5%). Prior antibiotic use (aOR 3.01; 95% CI 1.47–6.37), exposure to two agents (aOR 4.13; 95% CI 1.94–8.89) and use of AWaRe Access antibiotics (aOR 22.2; 95% CI 5.83–101) were strongly associated with colonization. Longer hospitalization and central catheter use were also associated with greater colonization odds, whereas total parenteral nutrition showed a protective association. In the sub-analysis, Access, Watch, and Reserve antibiotics were independently associated with ESBL-E and E-CPE colonization. **Conclusions:** Among transferred neonates, prior antibiotic exposure, particularly AWaRe-classified agents, showed the strongest association with intestinal colonization by Enterobacteriaceae, including ESBL-E/CPE phenotypes. Strengthening antimicrobial stewardship in referral facilities and implementing risk-based screening at admission may help reduce colonization and limit the spread of resistance.

## 1. Introduction

Neonatal sepsis remains one of the principal causes of morbidity and mortality in neonatal units, accounting for nearly 50% of all neonatal deaths worldwide [1]. This situation is particularly pronounced in low- and middle-income countries, where the incidence of confirmed sepsis is 46.9 per 1000 live births, leading to a mortality rate of 0.83 per 1000 neonate days [2]. Antimicrobial resistance (AMR) has become a global public health crisis [3,4,5], with significant clinical challenges in neonatal units [6]. This issue has intensified in low-middle income countries (LMICs), where the increased prevalence of resistant bacteria diminishes the efficacy of empirically prescribed antibiotics for neonatal sepsis, rendering these essential treatments ineffective against common pathogens [7]. This highlights the necessity of assessing the risks associated with sepsis, the acquisition of resistant bacteria, and the development of effective targeted interventions for their prevention and control.

In this context, a significant risk factor for late-onset neonatal sepsis is prior intestinal colonization by Enterobacteriaceae [8]. During hospitalization, several factors contribute to the establishment and expansion of this colonization, including low birth weight, use of invasive medical devices, exposure to antimicrobial therapy, and the severity of the underlying critical illness [9]. Colonization by antimicrobial-resistant phenotypes such as ESBL-E or E-CPE represents an additional concern because of their association with worse clinical outcomes [10].

Interhospital transfers (IHT) are a fundamental component of neonatal care across all health systems. However, the epidemiological risks associated with these transfers are particularly pronounced in settings with limited resources. Modeling studies have shown that lower-acuity peripheral units can become sources of bacterial colonization owing to prior antibiotic exposure and suboptimal infection control practices. As a result, transferred neonates may act as conduits for the dissemination of resistant strains into tertiary referral centers [11]. Level IV neonatal intensive care units (NICUs) in LMICs are particularly affected, as they receive critically ill neonates who often arrive with histories of prolonged hospitalization, multiple invasive procedures, and extensive broad-spectrum antibiotic exposure [7]. This constellation of factors creates a substantial likelihood that neonates admitted to referral units are already colonized by highly resistant Enterobacteriaceae.

Understanding the risk factors that predispose referred neonates to intestinal colonization is particularly important in LMICs, where neonatal referral pathways, limited stewardship infrastructure, and heterogeneous infection control practices may amplify the acquisition and dissemination of antimicrobial resistant organisms. We hypothesized that prior antibiotic exposure, together with other early life clinical and care-related factors, would be independently associated with an increased likelihood of intestinal Enterobacteriaceae colonization upon admission to a high-complexity neonatal unit. Therefore, the objective of this study was to determine the association between early life risk factors, including the type and burden of prior antibiotic use, and intestinal *Enterobacteriaceae* colonization in neonates transferred to a level IV referral unit in low-income and middle-income countries.

## 2. Results

A total of 435 neonates who underwent IHT from peripheral units were included in the study. The cohort comprising 87 cases (colonized) and 348 controls (non-colonized). Among the colonized infants, *Klebsiella pneumoniae* was the most frequently isolated microorganism (53.6%), followed by *Escherichia coli* (19.5%). Notably, 46.0% of colonized neonates (n = 40) harbored *Enterobacteriaceae* isolates with resistant phenotypes (ESBL-E or E-CPE).

The clinical and demographic characteristics of the study population are summarized in Table 1. Cases had a lower birth weight (*p* < 0.001) and a lower median Ballard score at admission (36.0 vs. 37.0 weeks; *p* < 0.001). Compared with controls, colonized neonates also had a markedly longer median duration of hospitalization prior to transfer (15 vs. 4 days; *p* < 0.001) and longer catheter use (8 vs. 0 days; *p* < 0.001).

Prior antibiotic exposure was more prevalent among cases (70.0% vs. 37.0%; *p* < 0.001), including higher exposure to Watch (32.0% vs. 9.5%) and Access (21.0% vs. 1.1%) agents, according to the WHO AWaRe classification.

All three logistic regression models consistently identified prior antibiotic exposure and days of central catheter use as the strongest variables associated with intestinal Enterobacteriaceae colonization upon admission. In the first model, using any prior antibiotic exposure as the main explanatory variable (Table 2), previous antibiotic use was associated with three-fold higher odds of colonization (aOR = 3.01; 95% CI: 1.47–6.37; *p* = 0.004). Additionally, compared with oral feeding, both mixed feeding (aOR = 0.24; 95% CI: 0.07–0.70; *p* = 0.008) and total parenteral nutrition (aOR = 0.30; 95% CI: 0.10–0.78; *p* = 0.013) were associated with significantly lower odds of colonization.

The second model evaluated the dose–response relationship according to the number of antibiotic agents used before admission (Table 3). Relative to no antibiotic exposure, the use of two agents (aOR = 4.13; 95% CI: 1.94–8.89; *p* < 0.001) or more than two agents (aOR = 3.73; 95% CI: 1.22–11.0; *p* = 0.018) demonstrated the strongest association with colonization.

The model based on the WHO AWaRe classification (Table 4) showed the strongest association. Exposure to Access agents was associated with markedly increased odds of colonization (aOR = 22.2; 95% CI: 5.83–101; *p* < 0.001). Prior exposure to watching agents also conferred a substantially increased risk (aOR = 4.08; 95% CI: 1.43–11.3; *p* = 0.007). By contrast, exposure to Reserve agents was not statistically significant in the adjusted analysis (aOR = 1.69; 95% CI: 0.70–3.93; *p* = 0.200).

Across all models, cumulative days of central catheter use were consistently associated with higher odds of colonization, whereas feeding type showed an inverse association in the adjusted analyses.

Finally, the sub-analysis evaluating the odds of colonization with resistant Enterobacteriaceae phenotypes (ESBL-E and E-CPE) upon admission to the referral NICU (Table 5) showed markedly increased odds among neonates exposed to antibiotics from any AWaRe category. The highest association was observed in the Access group (aOR = 49.82; 95% CI: 11.47, 216.39; *p* < 0.001), followed by the Watch group (aOR = 14.31; 95% CI: 3.70, 55.25; *p* < 0.001), and the Reserve group (aOR = 5.25; 95% CI: 1.34, 20.51; *p* = 0.017).

## 3. Discussion

This study evaluated the relationship between early life antibiotic exposure and intestinal colonization by *Enterobacteriaceae*, a clinically relevant event that precedes antimicrobial resistance and invasive infection among neonates referred from lower-complexity units to a Level IV neonatal center in a Low- and Middle-Income Country (LMIC). Across the three complementary analytical approaches, prior antibiotic exposure was strongly and consistently associated with intestinal colonization upon admission. Notably, the magnitude of the association was greatest when antibiotic exposure was categorized using the WHO AWaRe framework with Access group antibiotics. In a dedicated secondary analysis, prior antibiotic exposure was associated with colonization by resistant *Enterobacteriaceae*. In parallel, cumulative days of hospitalization and central catheter use emerged as additional non-modifiable, but highly informative factors associated with colonization risk. Together, these findings highlight the key clinical and system-level drivers of early intestinal colonization in referred neonates and provide actionable stewardship-oriented evidence to inform infection prevention strategies within neonatal referral pathways. Intestinal colonization by *Enterobacteriaceae* is a well-recognized precursor of late-onset sepsis and a major determinant of neonatal vulnerability to multidrug-resistant (MDR) infections [9,12]. Understanding how early life antibiotic exposure shapes colonization dynamics is especially critical in LMIC settings, where fragmented referral networks, constrained stewardship infrastructure, and high antibiotic pressure converge to create an environment conducive to MDR transmission.

The AWaRe framework, established by the WHO Expert Committee as a stewardship tool to guide rational prescriptions based on antimicrobial spectrum, ecological impact, and therapeutic role [13], has been increasingly adopted as a quality indicator for monitoring antibiotic prescription practices across clinical settings [14]. Our findings extend the application of this framework to the neonatal referral context, suggesting that AWaRe-defined antibiotic exposure before admission can serve as a meaningful marker associated with an increased risk of intestinal colonization by both susceptible and resistant *Enterobacteriaceae*.

Interestingly, in our cohort, exposure to antibiotics classified within the Access group showed the strongest association with intestinal colonization, a pattern not typically reported in neonatal settings. Rather than contradicting the AWaRe framework, this finding may reflect contextual factors and potentially suboptimal prescribing practices in referral facilities. In neonatal care, Access antibiotics are often the most frequently prescribed agents; they are initiated early in life, commonly used empirically, and frequently continued for prolonged periods, sometimes in the absence of timely microbiological confirmation or structured de-escalation strategies [15].

It is important to emphasize that these associations do not imply that Access agents are intrinsically more likely to select for resistant *Enterobacteriaceae*. Instead, the elevated odds observed in our cohort likely reflect how these drugs are deployed early in life, widely across clinical scenarios, administered empirically, and often continued without culture confirmation or planned de-escalation. In this context, the Access category functions not as a biological driver of colonization risk but as a proxy marker of prescribing patterns that create repeated or prolonged selection pressure. When used as intended, short empiric courses with timely reassessment, Access agents remain the safest option within the AWaRe hierarchy, consistent with the WHO stewardship principles [14].

This unexpected association between Access to antibiotics and colonization requires further contextual consideration. In many LMIC neonatal referral networks, stewardship implementation remains variable and is often limited to tertiary centers. While structured antimicrobial stewardship programs in high-complexity NICUs have demonstrated meaningful reductions in antibiotic duration and improved rational use of AWaRe Access agents [16], most of the neonatal antibiotic exposure in our population occurred before transfer, within lower-complexity facilities, where stewardship infrastructure is less developed.

Consistent with recent multicenter reports, outborn neonates are frequently started on antibiotics for unconfirmed early onset sepsis or prophylactic coverage, with Access to antibiotics being predominant [17]. These prescribing patterns are driven not only by resource constraints, but also by behavioral and cognitive factors documented in LMIC contexts. Clinicians often initiate treatment without microbiological confirmation, do not obtain cultures, and rarely define reassessment timelines, resulting in prolonged empirical therapy despite limited clinical evidence [18,19].

Beyond structural limitations, prescriber attitudes also play a central role. Studies describe persistent gaps in diagnostic confidence and a strong risk-aversion bias, whereby Access antibiotics are perceived as “safer” or “milder” options relative to Watch or Reserve agents [20]. This perception leads to their early and widespread use, even when infection is not clinically evident, reinforcing a pattern of antibiotic exposure that paradoxically increases the selective pressure. In this context, our finding that Access agents were associated with colonization likely reflects their overuse as default therapy rather than a failure of the AWaRe framework itself.

The pattern of antibiotic use in these facilities likely contributed to these findings. In our cohort, Reserve (24.8%) and Watch (14.0%) antibiotics predominated, resulting in an Access/Watch ratio <1, an indicator of suboptimal prescription and a known marker of increased selective pressure and resistance risk, particularly in pediatric populations [21].

Together, these system- and behavior-level drivers support the interpretation that the greatest opportunity for intervention lies upstream of the NICU admission. Strengthening antimicrobial stewardship, expanding access to diagnostics, and embedding decision support guidance in lower-complexity neonatal units may help reduce inappropriate Access to antibiotics and mitigate early colonization before transfer.

Within the context of early and cumulative antimicrobial exposure, our findings align with prior evidence suggesting that antibiotic pressure, particularly when prolonged, is associated with a higher risk of intestinal colonization with resistant organisms [22]. Mechanistic data further supported these associations, indicating that selective antibiotic pressure may facilitate the expansion and persistence of resistant strains [23,24], partly through increased horizontal gene transfer in environments with high antimicrobial exposure [25]. Since antibiotic use is considered one of the strongest correlates of neonatal intestinal colonization patterns, and colonization with Enterobacteriaceae has been linked to subsequent MDR acquisition and higher mortality in NICUs [12], the elevated colonization rates observed in our cohort, 46.0% of which involved ESBL-E and E-CPE phenotypes, are clinically meaningful. Although some confidence intervals were wide, reflecting the limited number of resistant isolates, the consistency of the associations across all AWaRe categories underscores their epidemiological and clinical relevance.

Despite these observations, the strength of the associations, particularly in the AWaRe-based models and resistant phenotype sub-analysis, should be interpreted with caution. These analyses involved smaller sample strata and a limited number of resistant isolates, thus creating the potential for sparse data bias and model instability. The wide confidence intervals observed in the Access category, especially in the resistant colonization model, likely reflect this imprecision rather than a true magnitude difference in the biological effects. Furthermore, although we evaluated antibiotic exposure continuously and tested alternative categorizations (including the number of days of treatment and continuous modeling of hospital stay and device use), these specifications did not outperform the selected models in terms of interpretability or stability. Therefore, the observed associations represent the most consistent specification available within the constraints of the dataset; however, residual uncertainty remains and should be acknowledged when extrapolating these findings.

The predominance of broad-spectrum agents provides a plausible explanation for the strong association observed between prior antibiotic exposure and E-ESBL/E-CPE colonization. Although Watch-and Reserve antibiotics are typically associated with higher rates of resistance emergence and mortality, the rational use of Access antibiotics, such as simple penicillin and amoxicillin, has been associated with reduced resistance-related mortality, reinforcing the intended role of the AWaRe framework in guiding neonatal antibiotic stewardship [18].

Finally, we found that both antibiotic exposure and use of two or more antibiotic agents were associated with increased odds of *Enterobacteriaceae* colonization. These findings highlight the role of the cumulative antibiotic burden as a key factor in colonization risk. This is consistent with reports indicating that cumulative exposure in neonates is associated with the disruption of intestinal microbiota development and increased vulnerability to resistant colonization [26,27]. Similarly, Ouedraogo et al. described a high prevalence of ESBL-E in LMIC neonatal units and reported that prior or prolonged exposure to broad-spectrum agents was associated with the selection and spread of resistant *E. coli* and *K. pneumoniae* [28]. Together, these data emphasize that stewardship efforts in neonatal referral networks should account not only for the type of antibiotic prescribed but also for the cumulative burden of exposure and support the concept that AWaRe-based monitoring may need to be operationalized not only within tertiary NICUs but also within referring facilities to reduce colonization risk prior to transfer.

Beyond antibiotic exposure, our findings indicate that markers of illness severity and care intensity, particularly prolonged hospitalization and central catheter use, were independently associated with intestinal colonization by Enterobacteriaceae. This aligns with previous reports showing that nosocomial factors and invasive devices increase neonatal exposure to resistant microorganisms and may facilitate their acquisition through cross-transmission and environmental contamination [29]. Ouedraogo et al. described the combination of broad-spectrum antibiotic use, extended hospital stay, and reliance on invasive devices as a critical triad linked to the emergence and persistence of resistant bacteria in neonatal settings [28]. Our results extend this concept to referred neonates who frequently arrive after prolonged care in lower-complexity facilities. Collectively, these observations suggest that stewardship strategies in neonatal referral networks must encompass not only prescribing practices but also rigorous device management and efforts to minimize avoidable hospitalizations.

The biological plausibility of these associations is well supported. Excessive or inappropriate antibiotic exposure during early life has been linked to the disruption of the developing intestinal microbiome, enabling the overgrowth and persistence of resistant *Enterobacteriaceae* [30,31]. In referred neonates, who frequently arrive after prolonged hospitalization and multiple procedures, these selective pressures are likely compounded by environmental exposure in the referring units, which may help explain the elevated colonization risk observed at admission.

Nutritional practices also appeared to influence colonization dynamics. In our cohort, total parenteral nutrition (TPN) was independently associated with a lower likelihood of intestinal colonization than was enteral feeding. A plausible explanation for this observation is the reduced use of enteral feeding tubes in infants receiving TPN. These devices are well-documented reservoirs for high bacterial loads, including ESBL-E, with up to 89% harboring >1000 CFU/mL, and more than half containing *Enterobacteriaceae* or *Staphylococcus aureus*, sometimes within the first 24 h of use [32,33]. Feeding tubes support bacterial adherence and proliferation and can introduce pathogenic *Enterobacteriaceae* into the gastrointestinal tract [34]. The demonstration of identical Random Amplified Polymorphic DNA profiles between tube isolates and intestinal microbiota further supports their role as vectors for microbial transfer within the NICU environments [35,36]. Therefore, these mechanisms may help explain the lower colonization rates observed in infants receiving TPN.

Although breastfeeding is generally associated with healthier microbiome development and reduced colonization by resistant bacteria [37], this protective pattern was not evident in our cohort, which likely reflects the small proportion of infants receiving human milk at the time of transfer.

Evidence describing the factors associated with intestinal colonization by ESBL-E and E-CPE among neonates requiring referral to highly complex NICUs remains limited. Our findings highlight IHT as a critical and often under-recognized factor associated with colonization risk. Previous studies in pediatric intensive care settings have shown that transferred patients are more likely to carry carbapenemase-producing organisms, whereas in Colombia, Díaz et al. reported that 16.3% of colonized patients were referred by other institutions [38]. More broadly, IHTs have been identified as important contributors to antimicrobial-resistant pathogen dissemination across healthcare networks, facilitating the movement of multidrug-resistant and extensively drug-resistant organisms across facilities, and influencing regional epidemiology [39].

Given that our NICU exclusively receives neonates from lower-complexity centers, systematic risk identification and strengthened infection prevention measures are essential. In our setting, all referred infants undergo rectal screening at admission and remain in isolation until the results guide clinical management. Among transferred neonates, the convergence of prolonged prior hospitalization, exposure to invasive devices, and substantial antibiotic exposure may create conditions associated with intestinal colonization by resistant Enterobacteriaceae. Recognizing these factors at admission is important for accurate risk stratification and the implementation of targeted infection control interventions.

The clinical relevance of these observations is supported by the well-established association between intestinal colonization and subsequent neonatal sepsis. Altered gut microbiota profiles in septic infants compared with healthy neonates [40], together with evidence from pediatric ICUs, show that admission screening and targeted prevention bundles have been associated with reductions in healthcare-associated infections, length of stay, and costs [41], highlighting the potential importance of proactive strategies in referred neonatal populations.

Taken together, our findings suggest that strengthening prevention strategies in tertiary NICUs receiving inter-facility transfers may be beneficial, including enhanced colonization surveillance, judicious use of invasive devices, and risk-based isolation approaches. Importantly, these results align with the need for a robust antimicrobial stewardship in neonatal referral networks. National frameworks such as Colombia’s Antimicrobial Optimization Program (PROA) offer a pathway toward improving prescription practices, reducing selective pressure, and limiting the spread of antimicrobial resistance across interconnected levels of care [42].

Although these results offer clinically relevant insights, their generalizability should be cautiously interpreted. Some key observations, including the predominance of Access agents, empiric initiation without microbiological confirmation, and referral-driven care fragmentation, likely reflect practice patterns and system constraints specific to our local context. However, the broader associations observed between early antibiotic exposure, cumulative care intensity, and colonization risk align with the findings reported across LMIC neonatal networks. Therefore, while prescription profiles may differ across countries and referral systems, the underlying mechanisms we describe are likely applicable to similar settings where limited stewardship capacity and interhospital transfer are common.

This study had several limitations that merit consideration. First, its retrospective design introduces the potential for information bias owing to incomplete or inconsistent documentation. To mitigate this risk, we conducted an exhaustive review of multiple institutional sources, including electronic medical records, laboratory databases, and paraclinical reports, to maximize data completeness and reduce misclassification.

Second, the analysis was conducted in a single tertiary referral NICU, which may limit the external generalizability. Nonetheless, this unit receives transfers from a diverse network of hospitals nationwide, offering a heterogeneous neonatal population and reflecting clinical realities comparable to those of other LMIC referral systems.

Third, antibiotic-resistant phenotypes were defined using standard phenotypic susceptibility testing rather than molecular confirmation. Although genotypic assays improve mechanistic resolution, CLSI- and EUCAST-guided phenotyping remain operational standard for clinical practice and are well-suited for assessing colonization risk in real-world neonatal care.

Residual confounding by indication is also possible. Clinically ill neonates are inherently more likely to receive antibiotics, undergo invasive procedures, and experience prolonged hospitalization, which are factors that are also linked to colonization risk. While we adjusted for multiple severity proxies (gestational age, birth weight, catheter days, nutritional route, and hospitalization duration), these variables may not fully capture baseline acuity, and no standardized illness severity score was consistently available. Accordingly, antibiotic exposure may partly function as a proxy for underlying illness severity, and associations should be interpreted cautiously.

Finally, the extremely high adjusted odds ratios observed in the resistant-colonization sub-analysis likely reflect sparse data bias arising from the small numbers of ESBL-E/CPE isolates within specific AWaRe strata. This exploratory model was not powered as a primary endpoint, and wide confidence intervals indicated imprecision. Therefore, these findings should be interpreted as hypothesis generating rather than definitive.

Despite these limitations, this study had several strengths. It examines a well-defined neonatal referral population using systematic admission screening, allowing accurate ascertainment of colonization at a uniform clinical time point. The study design incorporated a structured case–control framework with multivariable modeling to reduce confounding factors. The use of the WHO AWaRe framework provides a standardized and globally aligned method to quantify antibiotic exposure and interpret antimicrobial pressure. Together, these methodological choices enhance the internal validity and support the relevance of our findings to antimicrobial stewardship efforts in referral-based neonatal care systems, particularly in LMIC contexts.

## 4. Materials and Methods

The study protocol was approved by the ethics committees of the participating institutions (Min No. CEIC-0260-2022). This retrospective case–control study was conducted within a single Level IV high-complexity Neonatal Intensive Care Unit (NICU), which functions as a major referral center in low- and middle-income countries (LMIC). The study population comprised all critically ill neonates who were transferred (inter-hospital transfers) from peripheral neonatal units and subsequently admitted to the referral NICU between November 2013 and July 2021.

### 4.1. Case and Controls Definitions

Cases were defined as all referred neonates who exhibited intestinal colonization by *Enterobacteriaceae* confirmed through a positive rectal swab culture obtained immediately upon admission to the referral NICU. Controls were defined as neonates with a negative rectal swab culture for *Enterobacteriaceae* upon admission during the same study period.

All eligible colonized neonates were included in this study. Controls were randomly selected from the pool of non-colonized referred neonates admitted during the same study period using a fixed 1:4 case-to-control ratio to improve the statistical efficiency and precision.

### 4.2. Microbiological Methods and Definitions

Intestinal colonization screening was performed in all newborns immediately after admission. Sample collection involved a rectal swab introduced approximately half a centimeter into the anus using rotating motion. The samples were initially inoculated into an appropriate liquid enrichment medium and processed in a clinical laboratory using the HB&L system. This technology, based on light scattering, allows for rapid detection of bacterial growth and initial presumptive screening for microorganism detection (average time to positivity: 6.5 h). Following initial detection, samples were cultured on selective agar for Gram-negative bacilli to isolate and confirm the presence of Enterobacteriaceae colonies.

Antimicrobial susceptibility testing was performed according to the most recent Clinical and Laboratory Standards Institute (CLSI) and European Committee on Antimicrobial Susceptibility Testing (EUCAST) guidelines. The resistance phenotypes were defined as follows: 1. Extended-spectrum -lactamase (ESBL)-producing Enterobacteriaceae: isolates capable of hydrolyzing third- and fourth-generation cephalosporins and aztreonam while remaining susceptible to cephamycins (e.g., cefoxitin) and carbapenems. Phenotypic confirmation required a ≥3 two-fold reduction in MICor a ≥5 mm increase in the inhibition zone for cefotaxime and/or ceftazidime in the presence of clavulanate. 2. Carbapenemase-producing Enterobacteriaceae (CPE): isolates exhibiting reduced susceptibility to carbapenems (ertapenem >0.5 mg/L; meropenem or imipenem >2 mg/L), according to EUCAST screening criteria, followed by confirmatory testing for carbapenemase production.

### 4.3. Data Collection and Definition of Exposure

Clinical data were collected through a retrospective review of electronic medical records to characterize the study population and identify potential risk factors. Variables obtained upon admission included sex, gestational age (by Ballard, in weeks), and birth weight, recorded in grams and categorized for descriptive analyses as (categorized as <1500 g, 1500–2499 g, and ≥2500 g). Additionally, we recorded the admission weight (in grams) and cumulative number of days prior to transfer for hospital stay, days of catheter use, days on mechanical ventilation, and days on TPN. Nutritional factors such as the type of feeding route (oral, mixed feeding, or total parenteral nutrition) and whether the neonate received exclusive breastfeeding (yes/no) were also recorded.

For the primary exposure analysis, prior antibiotic exposure was defined as the administration of any systemic antibiotic documented before rectal swab collection. This exposure was analyzed in three distinct ways: 1. Antibiotic use: prior antibiotic use (yes/no); 2. Number of Antibiotics: The total number of distinct antibiotic agents used (categorized as None, Two, or >2); 3. AWaRe Classification: All antibiotics were categorized according to the 2024 World Health Organization Access, Watch, and Reserve (AWaRe) classification to assess the potential differential impact of antibiotic spectrum categories and evaluate patterns relevant to antimicrobial stewardship.

Although antibiotic exposure was defined strictly as therapy administered before rectal swab collection, the retrospective nature of the study does not allow the determination of the precise timing of intestinal colonization. Given that many neonates experienced prolonged hospitalization prior to transfer, colonization may have occurred before or concurrently with antibiotic exposure at the referring facilities. Therefore, while exposure preceded outcome measurement, the study design could not establish temporal causality, and the associations observed should be interpreted accordingly.

### 4.4. Statistical Analysis

Categorical variables were summarized as frequencies and percentages. Continuous variables were assessed for distributional normality using visual inspection and the Shapiro–Wilk test and were reported as mean (standard deviation [SD]) or median (interquartile range [IQR]), as appropriate. Baseline differences between cases and controls were evaluated using Student’s *t* test or Mann–Whitney U test for continuous variables and χ^2^ test or Fisher’s exact test for categorical variables.

The primary objective was to estimate the independent association between prior antibiotic exposure and intestinal *Enterobacteriaceae* colonization upon admission, using multivariable logistic regression. To capture the different dimensions of antibiotic exposure, three separate models were constructed: (1) any prior antibiotic use (yes/no), (2) number of distinct antibiotic agents used (categorized), and (3) the WHO AWaRe classification category.

Candidate variables were selected based on biological plausibility and statistical associations in univariate analyses (*p* < 0.20). Gestational age and birth weight were included a priori as key confounders given their clinical relevance and potential association with both antibiotic exposure and colonization risk. Multicollinearity was assessed using the Variance Inflation Factor (VIF), with a VIF of <5 considered acceptable. No variables were removed solely due to multicollinearity, as all candidate covariates demonstrated acceptable VIF values during model evaluation.

Model optimization was conducted using a manual stepwise elimination procedure, removing variables one at a time, starting with the least significant (highest *p*-value) and those contributing to high VIF. Adjustment was primarily guided by maintaining the stability of the Adjusted Odds Ratio (aOR) of primary exposure. We sought the most parsimonious model exhibiting the best balance between fit and complexity, as indicated by the lowest Akaike Information Criterion (AIC) and Bayesian Information Criterion (BIC) values. The final adjusted models for each exposure consistently included the following key nonmediating confounders: days of catheter use, days of hospital stay, and feeding route.

A dedicated sub-analysis was performed to identify the risk factors associated specifically with colonization by ESBL-E or E-CPE. For this analysis, the primary binary outcome was redefined as the presence of resistant colonization (1 = colonization by ESBL-E/E-CPE and 0 = colonization by other Enterobacteriaceae or absence of colonization). Given the critical role of the antibiotic spectrum in selecting resistance, the WHO AWaRe Classification was utilized as the primary exposure metric in the regression models for this subgroup.

We defined the outcome for the sub-analysis as the presence of intestinal colonization by ESBL-E or CPE at admission. For this purpose, neonates without resistant colonization (including those with no colonization or colonization by susceptible Enterobacteriaceae) were grouped in the reference category. This specification was selected to preserve statistical power and reflect the clinically relevant comparison of interest at NICU admission: carriage of resistant organisms versus absence of resistant colonization.

The results were reported as Adjusted Odds Ratios (aOR) with 95% confidence intervals (95% CI) and corresponding *p*-values. All statistical analyses were performed using the R programming language (version 4.4.1) within the RStudio integrated development environment (current version 2024.09.1 + 494).

## 5. Conclusions

In conclusion, among neonates transferred to a level-IV referral NICU, the factors most strongly associated with intestinal colonization by *Enterobacteriaceae*, including ESBL-E/CPE phenotypes, were related to prior antibiotic exposure in the originating institution. Exposure to antibiotics within the WHO AWaRe categories, particularly Access agents, as well as any prior use or exposure to multiple agents, showed the highest observed association with colonization risk.

Markers of care intensity, such as prolonged hospitalization and the use of invasive devices, were also associated with higher odds of colonization at admission, suggesting a multifactorial context in which antibiotic exposure and clinical interventions coexist in shaping colonization patterns in this high-risk population.

These findings add to the growing body of evidence regarding the risks associated with the neonatal referral system. Strengthening antimicrobial stewardship practices, implementing risk-based screening strategies, and optimizing device use at the time of admission may represent promising approaches for mitigating the burden of antimicrobial resistance in transferred neonates.

## Figures and Tables

**Table 1:** Baseline clinical characteristics of referred neonates at admission, stratified by Enterobacteriaceae colonization status.

Characteristic	Overall N = 435	Control N = 348	Case N = 87	*p*-Value
Sex, n (%)				0.40
Female	218 (50.0)	178 (51.0)	40 (46.0)	
Male	217 (50.0)	170 (49.0)	47 (54.0)	
Birth weight, n (%)				<0.001
≥2500 g	261 (60.0)	221 (63.5)	40 (46.0)	
1500–2499 g	115 (26.4)	90 (25.9)	25 (28.7)	
<1500 g	59 (13.6)	37 (10.6)	22 (25.3)	
Gestational Age by Ballard (weeks), Median (IQR)	37.0 (4)	37.0 (3)	36.0 (9)	<0.001 *
Admission weight (grams), Median (IQR)	2685 (1062)	2737 (940)	2423 (1455)	0.002 *
Days of Hospital Stay †, median (IQR)	5 (10)	4 (7)	15 (23)	<0.001 *
Days of Catheter Use †, median (IQR)	0 (2)	0 (0)	8 (20)	<0.001 *
Days on Total Parenteral Nutrition † median (IQR)	0.0 (0)	0.0 (0)	0.0 (5)	<0.001 *
Days on Mechanical ventilation †, median (IQR)	1.0 (3)	1.0 (0)	1.0 (20)	0.01 *
Type of Feeding Route, n (%)				0.37
Oral	273 (62.8)	224 (64.4)	49 (56.3)	
Mixed feeding	66 (15.2)	51 (14.7)	15 (17.2)	
Total parenteral nutrition	96 (22.1)	73 (21.0)	23 (26.4)	
Exclusive breastfeeding, n (%)				0.30
No	325 (75.0)	256 (74.0)	69 (79.0)	
Yes	110 (25.0)	92 (26.0)	18 (21.0)	
Antibiotic use, n (%)				<0.001
No	244 (56.0)	218 (63.0)	26 (30.0)	
Yes	191 (44.0)	130 (37.0)	61 (70.0)	
Number of antibiotic agents used, n (%)				<0.001
None	244 (56.1)	218 (62.6)	26 (30.0)	
Two	137 (31.5)	102 (29.3)	35 (40.0)	
>2	54 (12.4)	28 (8.1)	26 (30.0)	
AWaRe Classification, n (%)				<0.001
None	244 (56.1)	218 (63.0)	26 (30.0)	
Access	22 (5.1)	4 (1.1)	18 (21.0)	
Watch	61 (14.0)	33 (9.5)	28 (32.0)	
Reserve	108 (24.8)	93 (27.0)	15 (17.0)	

IQR: Interquartile range; TPN: Total parenteral nutrition. * Wilcoxon rank sum test. †: Cumulative days prior to transfer.

**Table 2:** Risk factors for intestinal *Enterobacteriaceae* colonization: unadjusted and adjusted Odds Ratios from a logistic regression model.

Characteristic	OR	95% CI	*p*-Value	aOR	95% CI	*p*-Value
Antibiotic use						
No	1.00 (ref.)			1.00 (ref.)		
Yes	3.93	2.39, 6.63	<0.001	3.01	1.47, 6.37	0.004
Days of Hospital Stay †	1.11	1.08, 1.15	<0.001	1.05	1.01, 1.09	0.020
Days of Catheter Use †	1.29	1.22, 1.39	<0.001	1.32	1.22, 1.47	<0.001
Type of Feeding Route						
Oral	1.00 (ref.)			1.00 (ref.)		
Mixed feeding	1.34	0.68, 2.54	0.40	0.24	0.07, 0.70	0.008
Total parenteral nutrition	1.44	0.81, 2.50	0.20	0.30	0.10, 0.78	0.013
Birth weight, n (%)						
≥2500 g	1.00 (ref.)			1.00 (ref.)		
1500–2499 g	1.21	0.66, 2.19	0.55	0.75	0.28, 1.85	0.53
<1500 g	4.92	2.74, 8.91	<0.001	1.21	0.24, 5.98	0.82
Gestational Age by Ballard, weeks †	0.86	0.75, 0.99	0.038	0.79	0.38, 1.62	0.52

Notes: N = 435. OR: Odds Ratio; CI: Confidence Interval. Adjusted OR is mutually adjusted for all other variables in the table. Gestational Age is presented as a continuous variable (per week increase). †: Cumulative days prior to admission.

**Table 3:** Risk factors for intestinal Enterobacteriaceae colonization: unadjusted and adjusted Odds Ratios based on the Number of Antibiotic Agents.

Characteristic	OR	95% CI	*p*-Value	aOR	95% CI	*p*-Value
Number of antibiotic agents used						
None	1.00 (ref.)			1.00 (ref.)		
Two	4.11	2.34, 7.30	<0.001	4.13	1.94, 8.89	<0.001
>2	8.61	4.42, 17.0	<0.001	3.73	1.22, 11.0	0.018
Days of Hospital Stay †	1.11	1.08, 1.15	<0.001	1.04	1.01, 1.08	0.034
Days of Catheter Use †	1.29	1.22, 1.39	<0.001	1.31	1.20, 1.45	<0.001
Type of Feeding Route						
Oral	1.00 (ref.)			1.00 (ref.)		
Mixed feeding	1.34	0.68, 2.54	0.40	0.20	0.06, 0.57	0.005
Total parenteral nutrition	1.84	1.03, 3.25	0.037	0.29	0.10, 0.75	0.015

Notes: N = 435. OR: Odds Ratio; CI: Confidence Interval. Adjusted OR is mutually adjusted for all other variables in the model. †: Cumulative days prior to admission.

**Table 4:** Risk factors for intestinal Enterobacteriaceae colonization: unadjusted and adjusted Odds Ratios based on the WHO AWaRe Classification of Antibiotics.

Characteristic	OR	95% CI	*p*-Value	aOR	95% CI	*p*-Value
AWaRe Classification						
None	1.00 (ref.)			1.00 (ref.)		
Access	37.7	13.0, 138	<0.001	22.2	5.83, 101	<0.001
Watch	7.11	3.74, 13.70	<0.001	4.08	1.43, 11.30	0.007
Reserve	1.35	0.67, 2.64	0.400	1.69	0.70, 3.93	0.200
Days of Hospital Stay †	1.11	1.08, 1.15	<0.001	1.03	1.00, 1.07	0.094
Days of Catheter Use †	1.29	1.22, 1.39	<0.001	1.30	1.20, 1.44	<0.001
Type of Feeding Route						
Oral	1.00 (ref.)			1.00 (ref.)		
Mixed feeding	1.34	0.68, 2.54	0.400	0.20	0.05, 0.60	0.007
Total parenteral nutrition	1.84	1.03, 3.25	0.037	0.30	0.10, 0.82	0.025

Notes: N = 435. OR: Odds Ratio; aOR: adjusted Odds Ratio; CI: Confidence Interval. Adjusted OR is mutually adjusted for all other variables in the model. †: Cumulative days prior to admission.

**Table 5:** Univariable and Multivariable Logistic Regression Analysis of Risk Factors for ESBL-E and E-CPE Resistance Colonization upon NICU Admission.

Characteristic	OR	95% CI	*p*-Value	aOR	95% CI	*p*-Value
AWaRe Classification						
None	1 (Ref.)			1 (Ref.)		
Access	96.4	26.13, 477.01	<0.001	49.82	11.47, 216.39	<0.001
Watch	31.04	9.92, 136.99	<0.001	14.31	3.70, 55.25	<0.001
Reserve	6.43	1.82, 29.80	0.007	5.25	1.34, 20.51	0.017
Days of Hospital Stay †	1.09	1.06, 1.12	<0.001	1.05	1.02, 1.08	<0.001

Notes: aOR: Adjusted Odds Ratio; CI: Confidence Interval. Adjusted OR is mutually adjusted for all other variables in the model. †: Cumulative days prior to admission.

## Data Availability

The data are not publicly available because of ethical and confidentiality restrictions. Anonymized data may be obtained from the corresponding author upon reasonable request and with the appropriate ethical approval.

## Abbreviations

The following abbreviations are used in this manuscript:

ESBL-E	Extended-Spectrum β-Lactamase-Producing Enterobacteriaceae
E-CPE	Enterobacteriaceae producing carbapenemases
WHO	World Health Organization
AMR	Antimicrobial resistance
LMIC	Low- and Middle-Income Country
IHT	Inter-hospital transfers
NICU	Neonatal intensive care unit
MDR	Multidrug-resistant
TPN	Total parenteral nutrition
PROA	Antimicrobial Optimization Program
CLSI	Clinical and Laboratory Standards Institute
EUCAST	European Committee on Antimicrobial Susceptibility Testing
